# Behaviour and stability of thermodilution signals in a closed extracorporeal circuit: a bench study

**DOI:** 10.1007/s10877-023-01018-0

**Published:** 2023-05-11

**Authors:** Elia J. Stanger, David C. Berger, Hansjörg Jenni, Kaspar F. Bachmann

**Affiliations:** 1 Department of Intensive Care Medicine, Inselspital, Bern University Hospital, University of Bern, Freiburgstrasse 3000, Bern, Switzerland; 2 Department of Cardiovascular Surgery, Inselspital, Bern University Hospital, University of Bern, Bern, Switzerland

**Keywords:** Extracorporeal membrane oxygenation, Cardiac output, Right ventricular function, Thermodilution

## Abstract

Thermodilution is the gold standard for cardiac output measurement in critically ill patients. Its application in extracorporeal therapy is limited, as a portion of the thermal indicator is drawn into the extracorporeal circuit. The behaviour of thermodilution signals in extracorporeal circuits is unknown. We investigated thermodilution curves within a closed-circuit and assessed the impact of injection volume, flow and distance on the behaviour of the thermodilution signals and catheter constants. We injected 3, 5, 7 and 10 ml of thermal indicator into a heated closed circuit. Thermistors at distances of 40, 60, 80, and 100 cm from the injection port recorded the thermodilution signals (at flow settings of 0.5, 1, 1.5, and 2 L/min). Area under the curve (AUC), rise time, exponential decay and catheter constants were analysed. Linear mixed-effects models were used to evaluate the impact of circuit flow, distance and injection volume. Catheter positioning did not influence AUC (78 injections). Catheter constants were independent of flow, injection volume or distance to the injection port. The distance to the injection port increased peak temperature and rise time and decreased exponential time constant significantly. The distance to the injection port did not influence catheter constants, but the properties of the thermodilution signal itself. This may influence measurements that depend on the exponential decay of the thermodilution signal such as right ventricular ejection fraction.

**Supplementary Information:**

The online version contains supplementary material available at 10.1007/s10877-023-01018-0.

## Introduction

Bedside measurement of cardiac output using thermodilution techniques in critically ill patients is a well-established concept, and still considered the clinical gold standard [1]. Transcardiac thermodilution uses an injection of a cold fluid bolus into a central vein with measurement of the resulting temperature difference further down-stream in the pulmonary artery [2, 3]. In transpulmonary thermodilution, the temperature difference caused by an injection in the right atrium is measured in a large systemic artery [4]. The Stewart-Hamilton equation states that the flow (I. E. cardiac output) is inversely proportional to the area under the temperature curve [3, 5]. Thermodilution also allows measurement of right ventricular ejection fraction and volumes by analysis of the exponential decay [6, 7]. In the setting of extracorporeal membrane oxygenation, thermodilution techniques have been shown not to work properly due to drainage of injectate into the extracorporeal circuit [6, 8]. Nevertheless, assessment of cardiac output during ECMO therapy may be of utmost importance for patient management [9, 10].

In a previous study, we have used a modified thermodilution technique to assess right ventricular function in the setting of veno-arterial ECMO [6]. In this study, we have introduced a thermistor into the ECMO inlet and have shown that catheter constants can be calibrated using the injections into the ECMO drainage with measurements of the signal. However, the effects of the distance between the injection port and the thermistor as well as the volume of injectate on the catheter constants and the behaviour of the thermodilution curve in extracorporeal setups are unknown. We therefore aim to assess the behaviour of thermodilution signals in a closed loop system with catheters at varying distances from the injection port. We hypothesize, that the distance between the injection port and thermistor as well as the injection volume have an impact on the properties of the thermodilution signals and we will assess whether the area under the curve (AUC) and calculated catheter constants are influenced by these changes in thermodilution signal properties.

## Methods

This study did not need approval from any ethics committee and was funded by internal resources of the Department of Intensive Care Medicine.

### Experimental setup

**Fig. 1:**
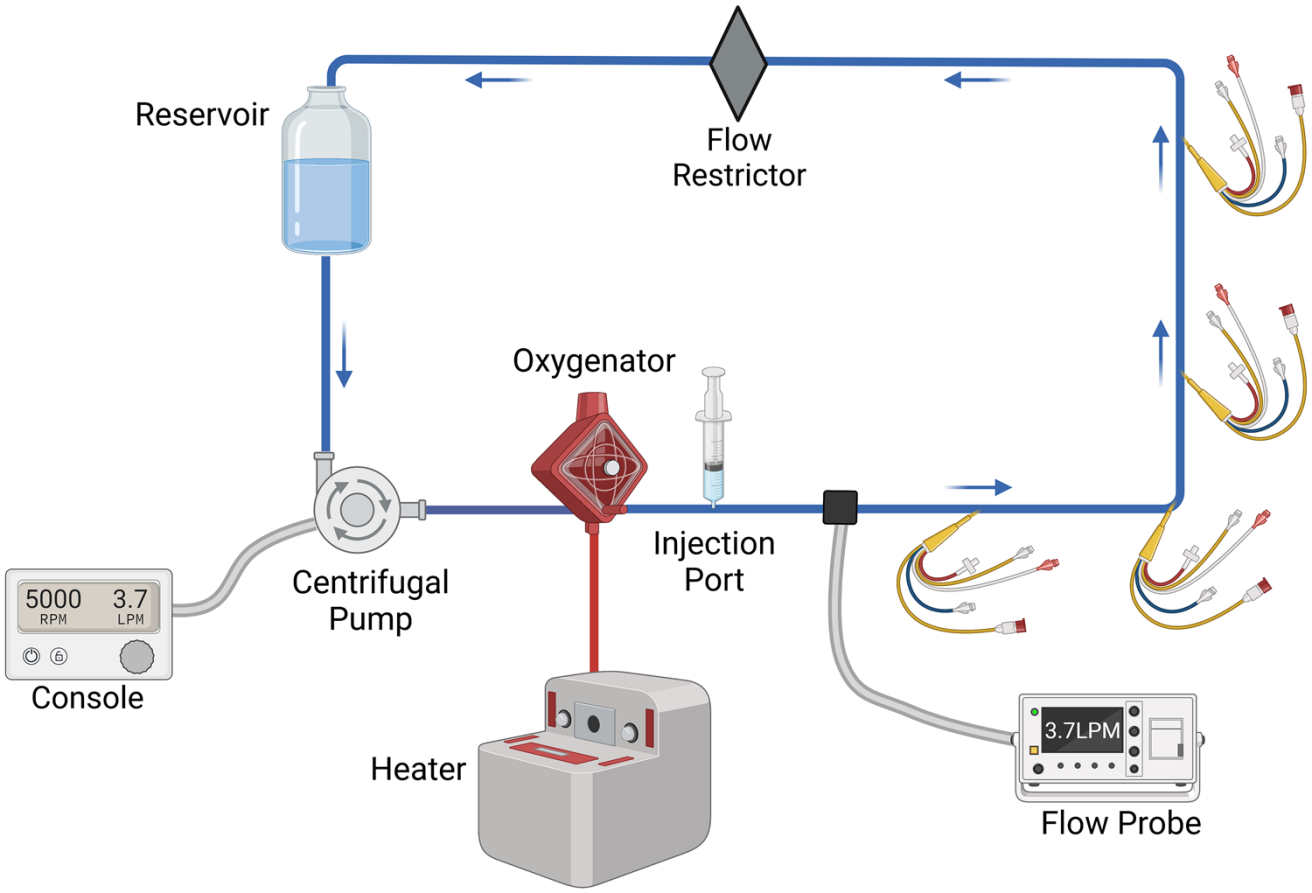
Experimental setup. Bends within the circuit are only in the illustration, the actual setup consisted of a round circuit without any sharp corners. Created with biorender.com

The circuit consisted of a reservoir (EL240 Blood Collection Reservoir, Medtronic plc, Dublin, Ireland), tubes (3/8" and right before and after oxygenator 1/4"), a rotation pump (Bio Console 560, Affinity CP AP40, Medtronic plc, Dublin, Ireland) and an oxygenator (QUADROX-I Paediatric Oxygenator, MAQUET, Hirrlingen, Germany), used for heating (HCV, Type 20–602, Jostra Fumedica, Muri, Switzerland, Fig. 1). The system was primed with 450 ml of lactated ringer’s solution and heated to 37 °C. An injection port was placed after the oxygenator. Four pulmonary artery catheters (131F7 Standard Four Lumen Catheter, Edwards Lifesciences, Irvine CA, US) were introduced at 40, 60, 80 and 100 cm after the injection port using a Y-introductory sheath (Y-Adapter 9,5 F, B.Braun Medical AG, Melsungen, Germany). A flow restrictor was used to accurately adjust flow settings as the resistance in the system was too low to regulate with pump speed only. The tube lengths were as following: 56 cm between pump and oxygenator, 173 cm between oxygenator and reservoir and 52 cm between reservoir and pump. An ultrasonic flow probe (TS410 Tubing Flow Module with a ME9PLX Flow sensor, Transonic Systems Inc., Ithaca NY, US) was attached to the tubing to achieve precise measurement of fluid flow.

### Experimental protocol and data acquisition

The experimental protocol consisted of a total of 80 injections into the circuit with simultaneous measurement of total flow and of the thermodilution signals from each catheter. 5 injections each were performed at 4 different flow settings (500, 100, 1500, 2000 ml/min) and 4 different injection volumes (3, 5, 7, 10 ml). The room temperature and thus temperature of the injectate was maintained at 23 °C. The pulmonary artery catheters were connected to either a Vigilance I (2 catheters, Edwards Lifescience, Irvine CA, US) or a Vigilance II (2 catheters, Edwards Lifescience, Irvine CA, US) with an analogue data output connected to an analog–digital converter board (BNC-2111, National Instruments, Austin TX, US). Data from the flow probe were collected with the same system. Room and circuit temperature were controlled manually after each set of 5 injections. Each injection triggered a data acquisition sequence of 30 s. Data acquisition was performed at a sample rate of 200 Hz using MatLab (v2022a, Mathworks, Natwick MA, US).

### Signal processing, outcomes and statistical analysis

All signals were visually inspected for artifacts and plausibility. Thermodilution signals were set manually to a baseline of zero at the end of each exponential decay to remove baseline drifts occurring after repeated bolus injections.

Temperature peaks were clipped due to a maximum output of 1 V (= 2 °C) by the vigilance devices at high injection volumes and low flows. Saturated signals were reconstructed by fitting a higher degree polynomial through the adjacent points of the saturated signal. The reconstructed signals were used for analysis and the following outcomes were calculated: 1) Area under the curve via the trapezoidal method. The signals were integrated over a sample rate of 100 Hz to enable comparison to our previous works [6]. 2) Peak of the signal using the signal maximum. 3) Start of the temperature rise using the differential of a smoothed signal (moving average window of 50 samples) and the rise time calculated as the time between rising point in temperature and the maximum of the signal. 4) The signal between the maximum and the temperature curve up to 0.3° was analysed using an exponential fit (nonlinear least squares) such that $f\left( x \right) = a*e^{bx}$. The coefficient b was determined as the time constant for the exponential decay of each thermodilution signal. 5) For each signal and injection we calculated a catheter constant “CC” such that: [6]

1 $$CC\; = \;\frac{Circuit\;Flow*AUC}{{Injection\;Volume*\left( {Circuit\;Temperature\; - \;Injection\;Temperature} \right)}}$$

Data are expressed as mean with standard deviation. Multiple linear mixed effect models with each manoeuvre (consisting of 5 injections) as random effect variables were used to analyse the impact of injection volume, circuit flow and catheter distance on different thermodilution properties. Effects of these models are reported using the intercept and the model estimates with 95% confidential intervals. Goodness of fit was assessed using adjusted R^2^ values. A two-tailed p-value < 0.05 was considered statistically significant.

## Results

**Fig. 2:**
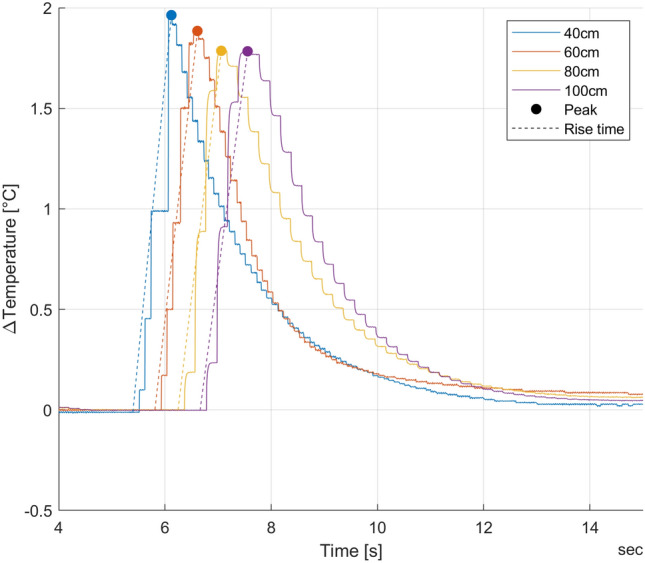
Thermodilution signals at 2000 ml/min circuit flow and an injection volume of 10 ml. The rise time (40 cm: 0.73 s, 60 cm: 0.80 s, 80 cm: 0.82 s, 100 cm: 0.90 s) and peak temperatures (40 cm: 1.96 °C, 60 cm: 1.88 °C, 80 cm: 1.79 °C, 100 cm: 1.78 °C) are graphically indicated for illustration purposes. The exponential coefficient for this injection were − 0.687 (40 cm), − 0.879 (60 cm), − 0.594 (80 cm) and − 0.649 (100 cm)

We included 78 injections into the final analysis. 2 injections at 1000 ml/min circuit flow and an injection volume of 3 ml were excluded due to insufficient signal quality. Each injection delivered 4 thermodilution signals (one per catheter), which resulted in analysis of 312 thermodilution signals (Fig. 2).

Mean circuit flow was well within protocol with flows of 502.2 ± 3.7 ml/min, 1003.8 ± 16.7 ml/min, 1504.0 ± 6.2 ml/min and 2002.1 ± 4.2 ml/min for each target flow setting. Injected volumes were according to protocol. Table 1 shows all data for each flow setting and injection volume as mean with standard deviation.

**Table 1:** Data expressed as mean and standard deviation according to different flow settings, injection volumes and catheter positions

Flow [ml/min]	Injection Volume [ml]	Peak temperature [°C]				Rise time [s]				Exponential decay coefficient			
		40 cm	60 cm	80 cm	100 cm	40 cm	60 cm	80 cm	100 cm	40 cm	60 cm	80 cm	100 cm
497.7 ± 1.0	3 ± 0	1.1 ± 0.0	0.9 ± 0.0	0.8 ± 0.0	0.8 ± 0.0	1.7 ± 0.1	1.8 ± 0.1	2.4 ± 0.2	2.6 ± 0.1	− 0.292 ± 0.028	− 0.323 ± 0.016	− 0.239 ± 0.018	− 0.222 ± 0.020
500.9 ± 1.5	5 ± 0	1.9 ± 0.0	1.5 ± 0.0	1.4 ± 0.0	1.3 ± 0.0	1.9 ± 0.1	2.0 ± 0.1	2.5 ± 0.1	2.8 ± 0.0	− 0.292 ± 0.013	− 0.282 ± 0.019	− 0.247 ± 0.024	− 0.228 ± 0.016
503.1 ± 0.6	7 ± 0	2.6 ± 0.1	2.1 ± 0.0	2.0 ± 0.0	1.9 ± 0.0	2.1 ± 0.1	2.2 ± 0.1	2.7 ± 0.1	3.1 ± 0.1	− 0.288 ± 0.006	− 0.233 ± 0.008	− 0.245 ± 0.008	− 0.233 ± 0.011
507.1 ± 1.2	10 ± 0	3.4 ± 0.4	2.6 ± 0.1	2.6 ± 0.1	2.5 ± 0.1	2.4 ± 0.1	2.4 ± 0.1	2.9 ± 0.1	3.2 ± 0.1	− 0.273 ± 0.024	− 0.207 ± 0.020	− 0.231 ± 0.014	− 0.220 ± 0.010
991.1 ± 19.1	3 ± 0	0.9 ± 0.1	0.8 ± 0.0	0.8 ± 0.1	0.8 ± 0.0	0.9 ± 0.1	1.0 ± 0.1	1.3 ± 0.0	1.2 ± 0.0	− 0.412 ± 0.030	− 0.556 ± 0.048	− 0.368 ± 0.065	− 0.369 ± 0.042
1015.5 ± 18.7	5 ± 0	1.3 ± 0.0	1.3 ± 0.0	1.2 ± 0.1	1.2 ± 0.0	0.9 ± 0.0	1.1 ± 0.1	1.3 ± 0.1	1.5 ± 0.1	− 0.433 ± 0.039	− 0.605 ± 0.032	− 0.405 ± 0.034	− 0.428 ± 0.027
992.9 ± 11.3	7 ± 0	1.9 ± 0.1	1.8 ± 0.1	1.6 ± 0.1	1.7 ± 0.1	1.1 ± 0.1	1.2 ± 0.1	1.6 ± 0.1	1.5 ± 0.1	− 0.429 ± 0.030	− 0.555 ± 0.053	− 0.403 ± 0.020	− 0.426 ± 0.024
1010.6 ± 5.1	10 ± 0	2.5 ± 0.1	2.4 ± 0.1	2.2 ± 0.0	2.3 ± 0.0	1.3 ± 0.0	1.4 ± 0.1	1.6 ± 0.1	1.6 ± 0.1	− 0.388 ± 0.030	− 0.475 ± 0.040	− 0.385 ± 0.020	− 0.412 ± 0.020
1503.6 ± 2.9	3 ± 0	0.7 ± 0.0	0.7 ± 0.0	0.6 ± 0.1	0.7 ± 0.0	0.7 ± 0.0	0.8 ± 0.1	0.9 ± 0.1	1.0 ± 0.0	− 0.463 ± 0.067	− 0.648 ± 0.043	− 0.450 ± 0.026	− 0.465 ± 0.021
1511.6 ± 1.1	5 ± 0	1.1 ± 0.1	1.1 ± 0.1	1.1 ± 0.1	1.1 ± 0.0	0.7 ± 0.1	0.8 ± 0.1	1.0 ± 0.1	1.1 ± 0.1	− 0.574 ± 0.068	− 0.777 ± 0.077	− 0.518 ± 0.037	− 0.546 ± 0.027
1499.6 ± 7.9	7 ± 0	1.6 ± 0.1	1.6 ± 0.1	1.4 ± 0.1	1.5 ± 0.0	0.8 ± 0.1	0.9 ± 0.1	1.0 ± 0.0	1.1 ± 0.1	− 0.494 ± 0.067	− 0.680 ± 0.059	− 0.450 ± 0.023	− 0.513 ± 0.040
1501.3 ± 1.3	10 ± 0	2.1 ± 0.1	2.2 ± 0.0	1.8 ± 0.1	2.1 ± 0.0	1.0 ± 0.1	1.0 ± 0.0	1.1 ± 0.1	1.2 ± 0.1	− 0.488 ± 0.035	− 0.670 ± 0.074	− 0.445 ± 0.027	− 0.530 ± 0.031
2005.7 ± 1.3	3 ± 0	0.6 ± 0.0	0.5 ± 0.0	0.5 ± 0.0	0.5 ± 0.0	0.5 ± 0.1	0.7 ± 0.1	0.7 ± 0.0	0.8 ± 0.1	− 0.658 ± 0.090	− 0.744 ± 0.052	− 0.548 ± 0.056	− 0.554 ± 0.050
2000.1 ± 6.7	5 ± 0	1.0 ± 0.1	1.0 ± 0.1	0.9 ± 0.1	0.9 ± 0.1	0.6 ± 0.1	0.8 ± 0.0	0.8 ± 0.1	1.0 ± 0.1	− 0.605 ± 0.087	− 0.807 ± 0.097	− 0.553 ± 0.054	− 0.596 ± 0.059
1999.2 ± 1.0	7 ± 0	1.4 ± 0.1	1.4 ± 0.0	1.2 ± 0.0	1.3 ± 0.0	0.7 ± 0.0	0.8 ± 0.1	0.8 ± 0.0	0.9 ± 0.1	− 0.578 ± 0.090	− 0.766 ± 0.092	− 0.555 ± 0.041	− 0.582 ± 0.047
2003.4 ± 1.8	10 ± 0	2.0 ± 0.1	1.9 ± 0.0	1.8 ± 0.0	1.9 ± 0.1	0.8 ± 0.1	0.8 ± 0.0	0.9 ± 0.1	1.0 ± 0.1	− 0.588 ± 0.076	− 0.792 ± 0.095	− 0.554 ± 0.046	− 0.605 ± 0.043

Flow [ml/min]	AUC				Catheter constant			
	40 cm	60 cm	80 cm	100 cm	40 cm	60 cm	80 cm	100 cm
497.7 ± 1.0	524.3 ± 42.5	509.7 ± 52.6	499.3 ± 61.5	493.2 ± 46.5	5.8 ± 0.5	5.6 ± 0.6	5.5 ± 0.7	5.5 ± 0.5
500.9 ± 1.5	887.8 ± 51.3	822.4 ± 21.4	822.4 ± 59.3	812.6 ± 40.7	5.9 ± 0.4	5.5 ± 0.2	5.5 ± 0.4	5.4 ± 0.3
503.1 ± 0.6	1321.0 ± 101.2	1244.5 ± 69.7	1209.7 ± 30.2	1177.9 ± 23.0	6.3 ± 0.5	6.0 ± 0.3	5.8 ± 0.2	5.6 ± 0.1
507.1 ± 1.2	1806.0 ± 50.7	1698.6 ± 93.8	1699.3 ± 147.9	1710.8 ± 154.5	6.1 ± 0.2	5.7 ± 0.3	5.7 ± 0.5	5.8 ± 0.5
991.1 ± 19.1	299.3 ± 16.9	307.1 ± 42.7	363.5 ± 97.0	380.4 ± 119.4	6.6 ± 0.4	6.8 ± 1.0	8.0 ± 2.3	8.4 ± 2.8
1015.5 ± 18.7	405.4 ± 36.5	378.7 ± 30.3	427.4 ± 58.4	425.4 ± 56.5	5.5 ± 0.4	5.1 ± 0.4	5.8 ± 0.7	5.8 ± 0.7
992.9 ± 11.3	618.1 ± 73.8	614.2 ± 91.7	647.1 ± 73.2	661.8 ± 72.9	5.8 ± 0.7	5.8 ± 0.8	6.1 ± 0.6	6.3 ± 0.6
1010.6 ± 5.1	880.1 ± 86.7	856.5 ± 86.7	842.0 ± 40.0	852.0 ± 49.4	5.9 ± 0.6	5.8 ± 0.6	5.7 ± 0.2	5.7 ± 0.3
1503.6 ± 2.9	213.3 ± 49.9	204.1 ± 48.0	185.5 ± 41.6	186.7 ± 38.0	7.1 ± 1.7	6.8 ± 1.6	6.2 ± 1.4	6.2 ± 1.3
1511.6 ± 1.1	248.2 ± 39.2	260.3 ± 32.7	338.3 ± 46.7	334.4 ± 47.9	5.0 ± 0.8	5.2 ± 0.7	6.8 ± 0.9	6.7 ± 1.0
1499.6 ± 7.9	426.0 ± 38.8	409.4 ± 29.4	420.3 ± 37.3	431.2 ± 26.3	6.1 ± 0.6	5.8 ± 0.4	6.0 ± 0.6	6.2 ± 0.4
1501.3 ± 1.3	570.0 ± 50.4	551.7 ± 30.6	551.2 ± 39.1	563.1 ± 46.0	5.7 ± 0.5	5.5 ± 0.3	5.5 ± 0.4	5.6 ± 0.5
2005.7 ± 1.3	116.5 ± 47.9	116.5 ± 35.1	127.4 ± 21.7	125.1 ± 22.4	5.2 ± 2.1	5.2 ± 1.6	5.7 ± 1.0	5.6 ± 1.0
2000.1 ± 6.7	217.2 ± 51.8	215.6 ± 43.6	232.4 ± 34.6	227.8 ± 31.2	5.8 ± 1.4	5.7 ± 1.2	6.2 ± 0.9	6.1 ± 0.8
1999.2 ± 1.0	319.7 ± 62.1	311.6 ± 42.9	302.7 ± 39.0	316.8 ± 40.7	6.1 ± 1.2	5.9 ± 0.8	5.8 ± 0.7	6.0 ± 0.8
2003.4 ± 1.8	420.7 ± 58.3	411.8 ± 29.2	455.1 ± 21.6	447.6 ± 31.4	5.6 ± 0.8	5.5 ± 0.4	6.1 ± 0.3	6.0 ± 0.4

**Fig. 3:**
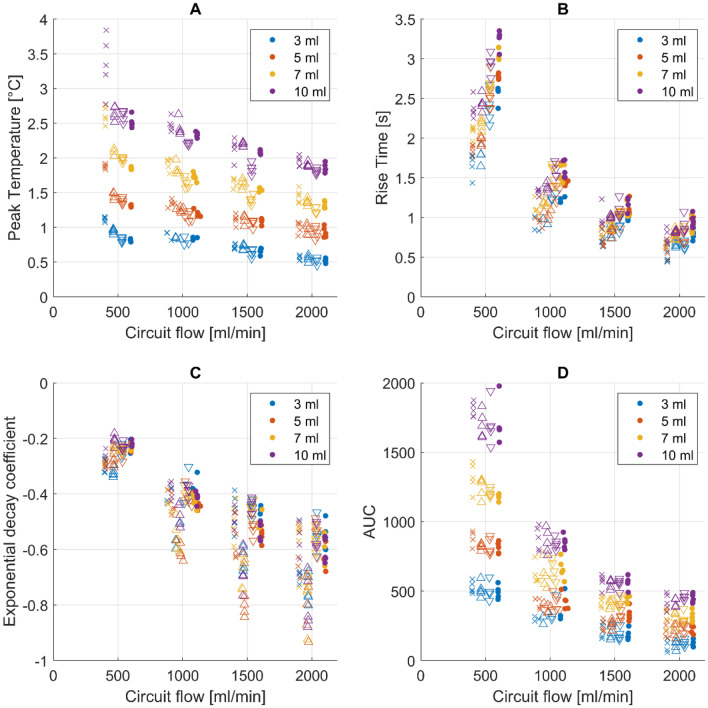
Scatter plot showing Peak Temperature (**A**), rise time (**B**), exponential decay coefficient (**C**) and area under the curve (**D**). Colors refer to the injection volume as indicated in the legend. Symbols depict different catheter positions: crosses refers to the catheter at 40 cm, upward-pointing triangles refer to the catheter at 60 cm, downward-pointing triangles refer to the catheter at 80 cm and dots refer to the catheter at 100 cm from the injection port. The position on the x-axis is jittered by ± 100 ml/min in order to visualize the data. Please refer to the Table 1 for true flow values

Peak temperature of the thermodilution signals decreased significantly with increasing distance from the injection port (Fig. 3A, Online supplement regression model 1, adjusted R^2^: 0.961). Additionally, higher circuit flow decreased, and injection volume increased peak temperature significantly. Multivariable regression models estimate an intercept of peak temperature of 0.81 ± 0.15 °C. The model estimated a decrease of − 0.44 ± 0.08 °C per 1 L/min flow change (p < 0.001) and an increase of 0.22 ± 0.02 °C per 1 mL increase in injection volume (p < 0.001). The catheter position, compared to the catheter at 40 cm away from the injection port, is estimated to impact the peak temperature by − 0.15 ± 0.04 °C at 60 cm (p < 0.001), − 0.26 ± 0.04 °C at 80 cm (p < 0.001) and − 0.23 ± 0.04 °C at 100 cm (p < 0.001).

The rise time of the thermodilution signals changes significantly depending on flow settings, injection volume as well as catheter position (Fig. 3B, online supplement model 2, adjusted R^2^: 0.956). The regression model estimates an intercept for rise time of 2.12 ± 0.43 s. Injection volume increased this by 0.05 ± 0.05 s per change of 1 ml (p = 0.037). Mean flow is estimated to change rise time by − 1.06 ± 0.22 s per change of 1 L/min flow (p < 0.001). The catheter position, compared to a distance of 40 cm from the injection port, is estimated to increase the rise time by 0.10 ± 0.05 s at 60 cm (p = 0.054), by 0.34 ± 0.05 s at 80 cm (p < 0.001) and by 0.47 ± 0. 05 s at 100 cm (p < 0.001).

**Fig. 4:**
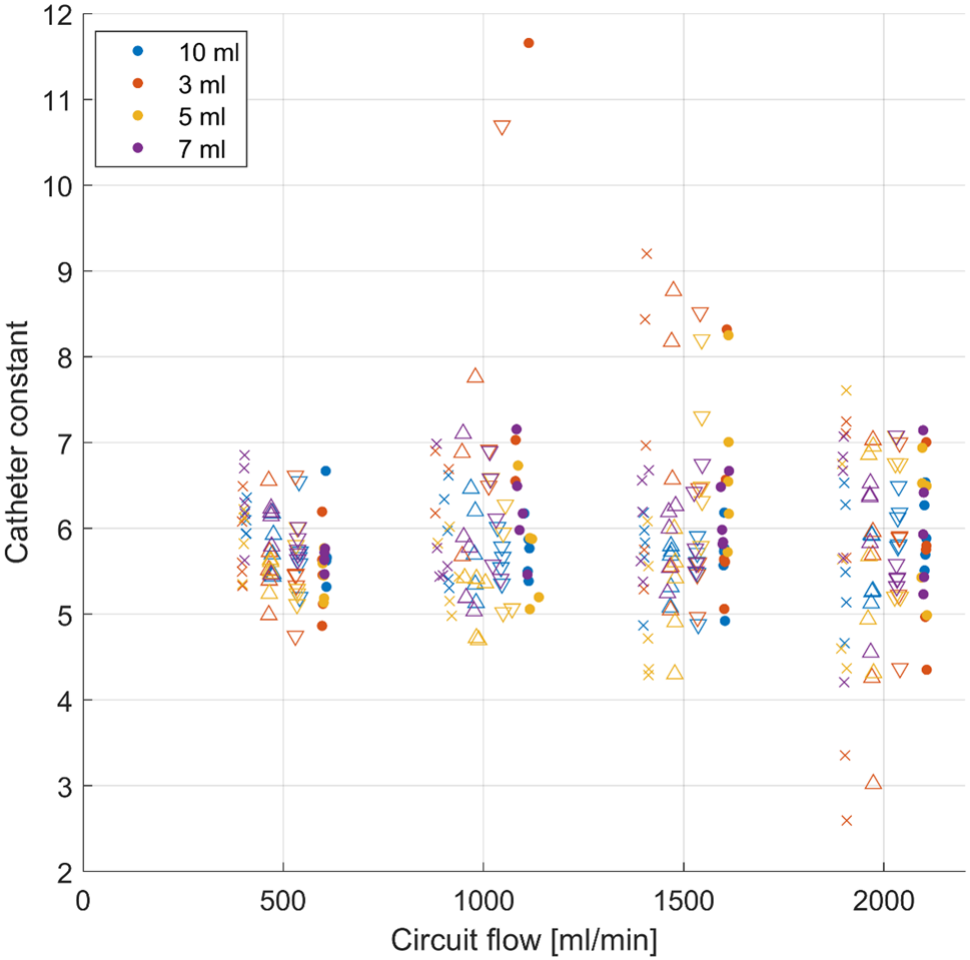
Scatter plot showing the calculated catheter constants for each injection. Colors refer to the injection volumes while symbols designate to different catheters as stated above. The flow values are jittered (± 100 ml/min) in order to visualize data. Please refer to Table 1 for true flow values

The catheter position as well as the circuit flow impacted the time constant of the exponential decay of the thermodilution signal significantly (Fig. 3C, online supplement model 3, adjusted R^2^: 0.875). Injection volume does not affect the exponential decay (p = 0.544). The model estimates an intercept for the time constant of − 0.156 ± 0.059. Mean flow was estimated to accelerate this by − 0.247 ± 0.030 per change of 1 L/min circuit flow (p < 0.001). The signal at 60 cm, compared to the signal at 40 cm, is estimated to show a faster exponential decay with a change of − 0.116 ± 0.019 (p < 0.001). Signals at 80 cm (increase of 0.041 ± 0.019, p < 0.001) and 100 cm (increase of 0.020 ± 0.019, p = 0.039) show a slower exponential decay.

The resulting AUC of each thermodilution curve is a function of injection volume and circuit flow, but not catheter position (Fig. 3D, online supplement model 4, adjusted R^2^: 0.981). The regression model estimates an intercept of 694.9 ± 294.9 °C*centiseconds. This estimate is decreased by − 530.2 ± 152.1 °C*centiseconds per change of 1 L/min circuit flow and increased by 88.0 ± 33.3 °C*centiseconds per increase of 1 ml of injection volume. Catheter position changes the AUC significantly, but only minimally (60 cm: − 23.3 ± 18.3 °C*centiseconds, p = 0.013 °C*centiseconds, 80 cm: − 11.3 ± 18.3 °C*centiseconds, p = 0.230, 100 cm: − 10.2 ± 18.3 °C*centiseconds, p = 0.276). Catheter constants, calculated from the AUC and ECMO circuit flow, remain independent of catheter position (p = 0.199), circuit flow (p = 0.996) and injection volume (p = 0.214, Fig. 4, online supplement model 6, adjusted R^2^: 0.127). The model estimated an intercept of 6.25 ± 0.74. Mean catheter constants with standard deviation for each catheter were 5.90 ± 0.99 (40 cm), 5.73 ± 0.85 (60 cm), 5.97 ± 0.90 (80 cm) and 6.00 ± 0.96 (100 cm). For all presented models, mean random effects were low. Details on each regression model can be found in the online supplement.

## Discussion

This study assessed the behaviour of thermodilution signals in extracorporeal circuits as a function of injection volume, distance to the injection port and circuit flow. Our data show, that catheter constants are independent of catheter positions and flow but the properties of the thermodilution signal itself change significantly with increasing distance from the injection port and by circuit flow. Signals closer to the injection port show a shorter rise time, higher peak temperature change and faster exponential decay. Catheter position heavily impacts the properties of the thermodilution signal. The regression models assess the different properties of the thermodilution curve in relation to circuit flow, catheter position as well as injection volume. Goodness of fit, expressed as the adjusted R^2^ value, is very high for these models, indicating that the relevant factors have been identified. Thermodilution is the clinical gold standard of cardiac output monitoring [3]. An error of approximately 15 to 20% has been estimated [11], which is in line with our results considering the standard deviation in calculated catheter constants (Fig. 4). In thermodilution, calculations of pulmonary blood flow are based upon a catheter constant that allow calculation of blood flow in L/min from $\frac{{Injection\,volume*\left( {body\,temperature\,-\,injection\,temperature} \right)}}{AUC}$. The catheter constant consists of two components such that c = K_1_ * K_2_ [3]_._ K_1_ is the product of specific heat and specific gravity of the injectate divided by the specific heat times the specific gravity of blood or medium [12]. In our in vitro setup, we can assume this factor to be very close to 1 as we injected ringer’s lactate into the circuit filled with ringer’s lactate with a temperature difference between injectate and circulating fluid of only 16 °C. Injection of saline or dextrose 5% into blood will increase K_1_ to slightly above 1 [13], as the specific heat capacity of blood is significantly lower than that of water or ringer’s lactate [14]. K_2_ is a computation constant that is dependent on catheter dead space, heat exchange in transit and injection rate [12]. Although the shape of the signal is altered significantly by catheter position, the resulting AUC stays constant in this study. From the assessed properties we conclude that the shorter rise time and faster exponential decay is compensated by a higher peak temperature, resulting in constant AUC values independent of catheter position. These constant AUCs result in a calculated catheter constant (formula 1) that is independent of catheter position. Circuit flow and injection volume do not impact the catheter constant, because it is scaled by these two variables (formula 1).

For pulmonary artery catheterization, catheter constants are provided by the manufacturer and are thought not to be influenced by blood or patient characteristics. However, applying thermodilution in the setting of extracorporeal circuits may require to recalibrate these constants in-vivo using the here described technique and data. Whether this improves cardiac output measurement compared to standard catheter constants should be assessed in future studies.

Cardiac output measurement during extracorporeal circulation is difficult and prone to error. Our study group has suggested approaches using either gas exchange measurements [15, 16] or modified thermodilution techniques [6]. In this modified thermodilution approach during VA-ECMO, we used an injection port and thermistor in the ECMO inlet. After calibration of individual catheter constants, we were able to calculate pulmonary blood flow using thermistors in the ECMO inlet as well as pulmonary artery, thus recording the entire signal produced by the injectate. In this VA-ECMO study in pigs, the calculated constants were between 4.5 and 5.3 and therefore slightly lower compared to the here presented results, which may be attributable to colder injectate (°4C) as well as injections into porcine blood rather than ringer’s lactate. We assumed that catheter constants were dependent on injection volume and corrected for this [6], but the data from this current study would suggest otherwise. Furthermore, it appears that in modified extracorporeal thermodilution techniques, the distance between the injection port and the thermistor is irrelevant as AUC remains constant. This may facilitate clinical practice, if such techniques are used further. Whether pulsatile versus continuous flow impacts catheter constants would need further assessment, but data from our previous studies suggest that the thermodilution curves are not affected by flow type [6]. While standard thermodilution in the setting of venovenous ECMO is currently not recommended, mainly due to indicator loss in both transcardiac and transpulmonary thermodilution [6, 8, 17], adapted techniques may allow quantification of recirculation [18] and ultimately maybe cardiac output.

Transcardiac thermodilution not only allows calculation of cardiac output but also enables estimates of right ventricular ejection fraction and in combination with stroke volume, derived from cardiac output, right ventricular filling volumes [19]. These calculations are based on the washout, e.g. the exponential decay of the thermodilution signal, such that the ejection fraction is calculated by dividing the signal height of beat n + 1 by the signal height of beat n during the washout phase [6, 19]. Compared to cardiac MRI and conductance catheter measurements, estimation of ventricular function using thermodilution has been proven to be of limited accuracy [7, 20]. Our results suggest that this exponential decay is a function of flow as well as distance to the injection port. Interestingly, the catheter at 60 cm has a significantly faster exponential decay compared to the other catheters. This effect seems to grow at increasing circuit flows (Fig. 3). This might indicate there still is a redistribution of injectate at this position, although rise time and peak temperature do not seem to be affected. This is in line with previous findings, where distance to the injectate port and catheter positioning impacted right ventricular indices [21, 22]. Our study confirms that changes in the exponential decay, depending on flow and catheter position, will inherently impact estimates of ventricular function. This should be considered when evaluating patients using thermodilution, particularly when injections are performed away from standard injections sites. Estimates of global end-diastolic volume and extravascular lung water derived from the exponential decay in transcardiac thermodilution methods may also be impacted by the distance to the injection site [4], but further study is needed to confirm this.

This study has limitations: 1) Thermodilution signals were clipped at 2 °C, which was an inherent limitation by the data acquisition equipment. We were able to correct for this using a polynomial fit, thus reconstructing the full thermodilution signal. 2) Catheters may have inherent precision errors [11], which led us to use the distance from the injection port as a categorical rather than continuous variable in multivariable regression models, allowing for detection of systematic bias or errors. 3) The introduced catheters could change the flow pattern and stir up turbulences. Using a colour solution, we determined that despite the introduction of catheters, the flow was laminar. However, small turbulences cannot completely be excluded and may limit the data interpretation.

In conclusion, our study shows that catheter constants for cardiac output calculations in extracorporeal circuits are independent of flow, injection volume and distance to catheter ports. This may be of importance for future studies assessing cardiac output in modified thermodilution techniques, particularly in the setting of veno-arterial of veno-venous ECMO. In vivo calibration of catheter constants appears to be reasonable and reliable. Clinicians should be careful when evaluating right heart indices through thermodilution, as these values are influenced by catheter position as well as blood flow.

## Supplementary Information

Below is the link to the electronic supplementary material.

Supplementary file1 (DOCX 25 kb)

## References

[1] Argueta EE, Paniagua D (2019). Thermodilution cardiac output: a concept over 250 years in the making. Cardiol Rev.

[2] Swan HJ, Ganz W, Forrester J, Marcus H, Diamond G, Chonette D (1970). Catheterization of the heart in man with use of a flow-directed balloon-tipped catheter. N Engl J Med.

[3] Reuter DA, Huang C, Edrich T, Shernan SK, Eltzschig HK (2010). Cardiac output monitoring using indicator-dilution techniques: basics, limits, and perspectives. Anesth Analg.

[4] Sakka SG, Reuter DA, Perel A (2012). The transpulmonary thermodilution technique. J Clin Monit Comput.

[5] Hamilton WF, Moore JW, Kinsman JM, Spurling RG (1928). Simultaneous determination of the pulmonary and systemic circulation times in man and of a figure related to the cardiac output. Am J Physiol..

[6] Bachmann KF, Zwicker L, Nettelbeck K, Casoni D, Heinisch PP, Jenni H (2020). Assessment of right heart function during extracorporeal therapy by modified thermodilution in a porcine model. Anesthesiology.

[7] Hein M, Roehl AB, Baumert JH, Rossaint R, Steendijk P (2009). Continuous right ventricular volumetry by fast-response thermodilution during right ventricular ischemia: head-to-head comparison with conductance catheter measurements. Crit Care Med.

[8] Russ M, Steiner E, Boemke W, Busch T, Melzer-Gartzke C, Taher M (2022). Extracorporeal membrane oxygenation blood flow and blood recirculation compromise thermodilution-based measurements of cardiac output. ASAIO J.

[9] Vieillard-Baron A, Matthay M, Teboul JL, Bein T, Schultz M, Magder S (2016). Experts’ opinion on management of hemodynamics in ARDS patients: focus on the effects of mechanical ventilation. Intens Care Med.

[10] Zante B, Berger DC, Schefold JC, Bachmann KF (2021). Dissociation of arterial oxygen saturation and oxygen delivery in VV-ECMO: the trend is your friend. J Cardiothorac Vasc Anesth.

[11] Yang X-X, Critchley LA, Joynt GM (2011). Determination of the precision error of the pulmonary artery thermodilution catheter using an in vitro continuous flow test rig. Anesth Analg.

[12] Nishikawa T, Dohi S (1993). Errors in the measurement of cardiac output by thermodilution. Can J Anaesth.

[13] Bootsma IT, Boerma EC, Scheeren TWL, de Lange F (2022). The contemporary pulmonary artery catheter. Part 2: measurements, limitations, and clinical applications. J Clin Monit Comput..

[14] Blake AS, Petley GW, Deakin CD (2000). Effects of changes in packed cell volume on the specific heat capacity of blood: implications for studies measuring heat exchange in extracorporeal circuits. Br J Anaesth.

[15] Bachmann KF, Haenggi M, Jakob SM, Takala J, Gattinoni L, Berger D (2020). Gas exchange calculation may estimate changes in pulmonary blood flow during veno-arterial extracorporeal membrane oxygenation in a porcine model. Am J Physiol Lung Cell Mol Physiol.

[16] Bachmann KF, Vasireddy R, Heinisch PP, Jenni H, Vogt A, Berger D (2021). Estimating cardiac output based on gas exchange during veno-arterial extracorporeal membrane oxygenation in a simulation study using paediatric oxygenators. Sci Rep.

[17] Herner A, Lahmer T, Mayr U, Rasch S, Schneider J, Schmid RM (2020). Transpulmonary thermodilution before and during veno-venous extra-corporeal membrane oxygenation ECMO: an observational study on a potential loss of indicator into the extra-corporeal circuit. J Clin Monit Comput.

[18] Cipulli F, Battistin M, Carlesso E, Vivona L, Cadringher P, Todaro S, et al. Quantification of recirculation during veno-venous extracorporeal membrane oxygenation: in vitro evaluation of a thermodilution technique. ASAIO J. 2022;68:184–9. 10.1097/MAT.0000000000001428 33788801

[19] Rapaport E, Wong M, Ferguson RE, Bernstein P, Wiegand BD (1965). Right ventricular volumes in patients with and without heart failure. Circulation.

[20] Hoeper MM, Tongers J, Leppert A, Baus S, Maier R, Lotz J (2001). Evaluation of right ventricular performance with a right ventricular ejection fraction thermodilution catheter and MRI in patients with pulmonary hypertension. Chest.

[21] Spinale FG, Zellner JL, Mukherjee R, Ferris SE, Crawford FA (1990). Thermodilution right ventricular ejection fraction. Catheter positioning effects. Chest.

[22] Cockroft S, Withington PS (1993). The measurement of right ventricular ejection fraction by thermodilution. A comparison of values obtained using differing injectate ports. Anaesthesia..

