# Supplementary material for: Behaviour and stability of thermodilution signals in a closed extracorporeal circuit: a bench study
Source: J Clin Monit Comput. 2023 May 11;37(4):1095–102. doi: 10.1007/s10877-023-01018-0 (PMC10371883; doi:10.1007/s10877-023-01018-0)
Supplement: Supplementary file 1 — Supplementary file1 (DOCX 25 kb) [file 10877_2023_1018_MOESM1_ESM.docx]

Behaviour of thermodilution signals in a closed extracorporeal circuit - a bench study

Elia J. Stanger, David Berger, Hansjörg Jenni, Kaspar F. Bachmann

Online supplement

Table of contents

[Multivariable regression model 1: peak temperature 2](#_Toc134200517)

[Multivariable regression model 2: Rise time 3](#_Toc134200518)

[Multivariable regression model 3: exponential decay 4](#_Toc134200519)

[Multivariable regression model 4: AUC 5](#_Toc134200520)

[Multivariable regression model 5: Catheter constants 6](#_Toc134200521)

# Multivariable regression model 1: peak temperature

**Linear mixed-effects model fit by ML**

**Model information:**

Number of observations 312

Fixed effects coefficients 6

Random effects coefficients 16

Covariance parameters 2

**Formula:**

PeakTemperature ~ 1 + InjectionVolume + MeanFlow + CatheterName + (1 | MeasurementNumber)

**Model fit statistics:**

AIC BIC LogLikelihood Deviance

-344.23 -314.28 180.11 -360.23

**Fixed effects coefficients (95% CIs):**

Name Estimate SE tStat DF pValue Lower Upper

{'(Intercept)' } 0.81205 0.078996 10.28 306 1.7356e-21 0.6566 0.96749

{'InjectionVolume' } 0.21952 0.0087582 25.064 306 3.8111e-76 0.20228 0.23675

{'MeanFlow' } -0.00043555 4.0368e-05 -10.79 306 3.2495e-23 -0.00051499 -0.00035612

{'CatheterName_Pulmi60cm' } -0.14505 0.020524 -7.0671 306 1.0739e-11 -0.18543 -0.10466

{'CatheterName_Pulmi80cm' } -0.26422 0.020524 -12.873 306 1.331e-30 -0.3046 -0.22383

{'CatheterName_Pulmi100cm'} -0.2268 0.020524 -11.05 306 4.1245e-24 -0.26718 -0.18641

**Random effects covariance parameters (95% CIs):**

Group: MeasurementNumber (16 Levels)

Name1 Name2 Type Estimate Lower Upper

{'(Intercept)'} {'(Intercept)'} {'std'} 0.085644 0.058237 0.12595

Group: Error

Name Estimate Lower Upper

{'Res Std'} 0.12817 0.11826 0.13892

# Multivariable regression model 2: Rise time

**Linear mixed-effects model fit by ML**

**Model information:**

Number of observations 312

Fixed effects coefficients 6

Random effects coefficients 16

Covariance parameters 2

**Formula:**

RiseTime ~ 1 + InjectionVolume + MeanFlow + CatheterName + (1 | MeasurementNumber)

**Model fit statistics:**

AIC BIC LogLikelihood Deviance

-221.78 -191.84 118.89 -237.78

**Fixed effects coefficients (95% CIs):**

Name Estimate SE tStat DF pValue Lower Upper

{'(Intercept)' } 2.125 0.22035 9.6437 306 2.1714e-19 1.6914 2.5586

{'InjectionVolume' } 0.05178 0.024748 2.0923 306 0.037235 0.0030825 0.10048

{'MeanFlow' } -0.0010575 0.00011396 -9.2795 306 3.2157e-18 -0.0012817 -0.00083322

{'CatheterName_Pulmi60cm' } 0.10167 0.023861 4.2608 306 2.7146e-05 0.054715 0.14862

{'CatheterName_Pulmi80cm' } 0.34269 0.023861 14.362 306 4.1304e-36 0.29574 0.38964

{'CatheterName_Pulmi100cm'} 0.46891 0.023861 19.652 306 3.5027e-56 0.42196 0.51586

**Random effects covariance parameters (95% CIs):**

Group: MeasurementNumber (16 Levels)

Name1 Name2 Type Estimate Lower Upper

{'(Intercept)'} {'(Intercept)'} {'std'} 0.25367 0.17821 0.36108

Group: Error

Name Estimate Lower Upper

{'Res Std'} 0.14901 0.13748 0.16151

# Multivariable regression model 3: exponential decay

**Linear mixed-effects model fit by ML**

**Model information:**

Number of observations 312

Fixed effects coefficients 6

Random effects coefficients 16

Covariance parameters 2

**Formula:**

ExponentialDecay ~ 1 + InjectionVolume + MeanFlow + CatheterName + (1 | MeasurementNumber)

**Model fit statistics:**

AIC BIC LogLikelihood Deviance

-830.72 -800.77 423.36 -846.72

**Fixed effects coefficients (95% CIs):**

Name Estimate SE tStat DF pValue Lower Upper

{'(Intercept)' } -0.15612 0.029848 -5.2305 306 3.1408e-07 -0.21485 -0.097387

{'InjectionVolume' } 0.001994 0.0032852 0.60695 306 0.54433 -0.0044705 0.0084584

{'MeanFlow' } -0.00024718 1.5126e-05 -16.342 306 1.3643e-43 -0.00027694 -0.00021741

{'CatheterName_Pulmi60cm' } -0.11592 0.0095149 -12.183 306 4.1761e-28 -0.13465 -0.097202

{'CatheterName_Pulmi80cm' } 0.041084 0.0095149 4.3178 306 2.1314e-05 0.022361 0.059806

{'CatheterName_Pulmi100cm'} 0.019763 0.0095149 2.077 306 0.038634 0.0010398 0.038486

**Random effects covariance parameters (95% CIs):**

Group: MeasurementNumber (16 Levels)

Name1 Name2 Type Estimate Lower Upper

{'(Intercept)'} {'(Intercept)'} {'std'} 0.031106 0.020631 0.046898

Group: Error

Name Estimate Lower Upper

{'Res Std'} 0.059421 0.054823 0.064404

# Multivariable regression model 4: AUC

**Linear mixed-effects model fit by ML**

**Model information:**

Number of observations 312

Fixed effects coefficients 6

Random effects coefficients 16

Covariance parameters 2

**Formula:**

AUC ~ 1 + InjectionVolume + MeanFlow + CatheterName + (1 | MeasurementNumber)

**Model fit statistics:**

AIC BIC LogLikelihood Deviance

3522.4 3552.3 -1753.2 3506.4

**Fixed effects coefficients (95% CIs):**

Name Estimate SE tStat DF pValue Lower Upper

{'(Intercept)' } 694.92 150.48 4.6181 306 5.7107e-06 398.82 991.02

{'InjectionVolume' } 88.027 16.983 5.1832 306 3.9695e-07 54.608 121.45

{'MeanFlow' } -0.53024 0.077618 -6.8314 306 4.5433e-11 -0.68298 -0.37751

{'CatheterName_Pulmi60cm' } -23.321 9.3559 -2.4927 306 0.013208 -41.731 -4.9109

{'CatheterName_Pulmi80cm' } -11.252 9.3559 -1.2027 306 0.23003 -29.662 7.158

{'CatheterName_Pulmi100cm'} -10.208 9.3559 -1.091 306 0.27612 -28.617 8.2025

**Random effects covariance parameters (95% CIs):**

Group: MeasurementNumber (16 Levels)

Name1 Name2 Type Estimate Lower Upper

{'(Intercept)'} {'(Intercept)'} {'std'} 175.16 123.59 248.25

Group: Error

Name Estimate Lower Upper

{'Res Std'} 58.427 53.905 63.329

# Multivariable regression model 5: Catheter constants

**Linear mixed-effects model fit by ML**

**Model information:**

Number of observations 312

Fixed effects coefficients 6

Random effects coefficients 16

Covariance parameters 2

**Formula:**

CatheterConstant ~ 1 + InjectionVolume + MeanFlow + CatheterName + (1 | MeasurementNumber)

**Model fit statistics:**

AIC BIC LogLikelihood Deviance

823.07 853.01 -403.53 807.07

**Fixed effects coefficients (95% CIs):**

Name Estimate SE tStat DF pValue Lower Upper

{'(Intercept)' } 6.245 0.37673 16.577 306 1.7365e-44 5.5037 6.9863

{'InjectionVolume' } -0.051381 0.041218 -1.2466 306 0.2135 -0.13249 0.029725

{'MeanFlow' } 1.0108e-06 0.00018959 0.0053313 306 0.99575 -0.00037205 0.00037407

{'CatheterName_Pulmi60cm' } -0.16505 0.1356 -1.2172 306 0.22448 -0.43187 0.10178

{'CatheterName_Pulmi80cm' } 0.077694 0.1356 0.57297 306 0.56709 -0.18913 0.34452

{'CatheterName_Pulmi100cm'} 0.099076 0.1356 0.73065 306 0.46555 -0.16775 0.3659

**Random effects covariance parameters (95% CIs):**

Group: MeasurementNumber (16 Levels)

Name1 Name2 Type Estimate Lower Upper

{'(Intercept)'} {'(Intercept)'} {'std'} 0.37921 0.23873 0.60236

Group: Error

Name Estimate Lower Upper

{'Res Std'} 0.84682 0.78108 0.9181
